# Primary Pulmonary Hydatid Cyst: A Rare Presentation

**DOI:** 10.7759/cureus.81575

**Published:** 2025-04-01

**Authors:** Sourav Sudan, Ninia Goyal, Lyluma Ishfaq, Navjot Kaur, Lakshmi Deepak Bethineedi

**Affiliations:** 1 Internal Medicine, Government Medical College Rajouri, Rajouri, IND; 2 Internal Medicine, Chirayu Medical College and Hospital, Bhopal, IND; 3 Medicine, Directorate of Health Services Kashmir, Srinagar, IND; 4 Medicine and Surgery, Government Medical College Patiala, Patiala, IND; 5 Medicine, Andhra Medical College, Visakhapatnam, IND

**Keywords:** albendazole antihelminthic, echinococcosis, echinococcosis granulosus, hydatid cyst in lung, pulmonary hydatid cyst

## Abstract

Hydatid disease is a zoonotic disease that is endemic in certain parts of India, especially rural India. Although most cases of hydatid disease affect the liver, it can occasionally affect other organs such as the spleen, lungs, spine, and intestines. We present a case of a hydatid cyst in the lung without any coexisting lesion in the liver.

## Introduction

Echinococcosis is a zoonotic infection caused by tapeworms belonging to the Echinococcus (E.) genus, with E. granulosus and E. multilocularis being the two most important species from a medical standpoint.

Hydatid cyst disease is considered endemic in India, and its incidence ranges from 1 to 200 per 100,000 people. Echinococcus can infect various organs, causing primary echinococcosis, and the daughter cysts from these primary locations may spread to other organ sites, resulting in secondary echinococcosis [1]. The most commonly affected organ in adults is the liver, followed by the lungs, with frequencies of 60% and 20-30%, respectively [2,3]. Due to the elastic structure of the lungs, hydatid cysts can attain giant sizes and be invasive to most of the lobes [4].

We report a case of a 30-year-old male who presented to the outpatient department of our center with a cough for two weeks and was found to have bilateral pulmonary hydatid cysts on further evaluation. This case is rare as this patient was diagnosed with primary pulmonary echinococcosis without any visible effects on the liver.

## Case presentation

A 30-year-old male presented to the outpatient department of our center with a cough for two weeks. The cough was productive and was not associated with fever, shortness of breath, weight loss, night sweats, or any other significant presentation.

A physical examination and auscultation of the lungs revealed no abnormalities. Vitals at the time of the first encounter were: blood pressure = 110/70 mmHg, pulse rate = 74 bpm, and SaO2 = 98%. The examination of other systems, including the cardiovascular system, was unremarkable.

**Figure 1:**
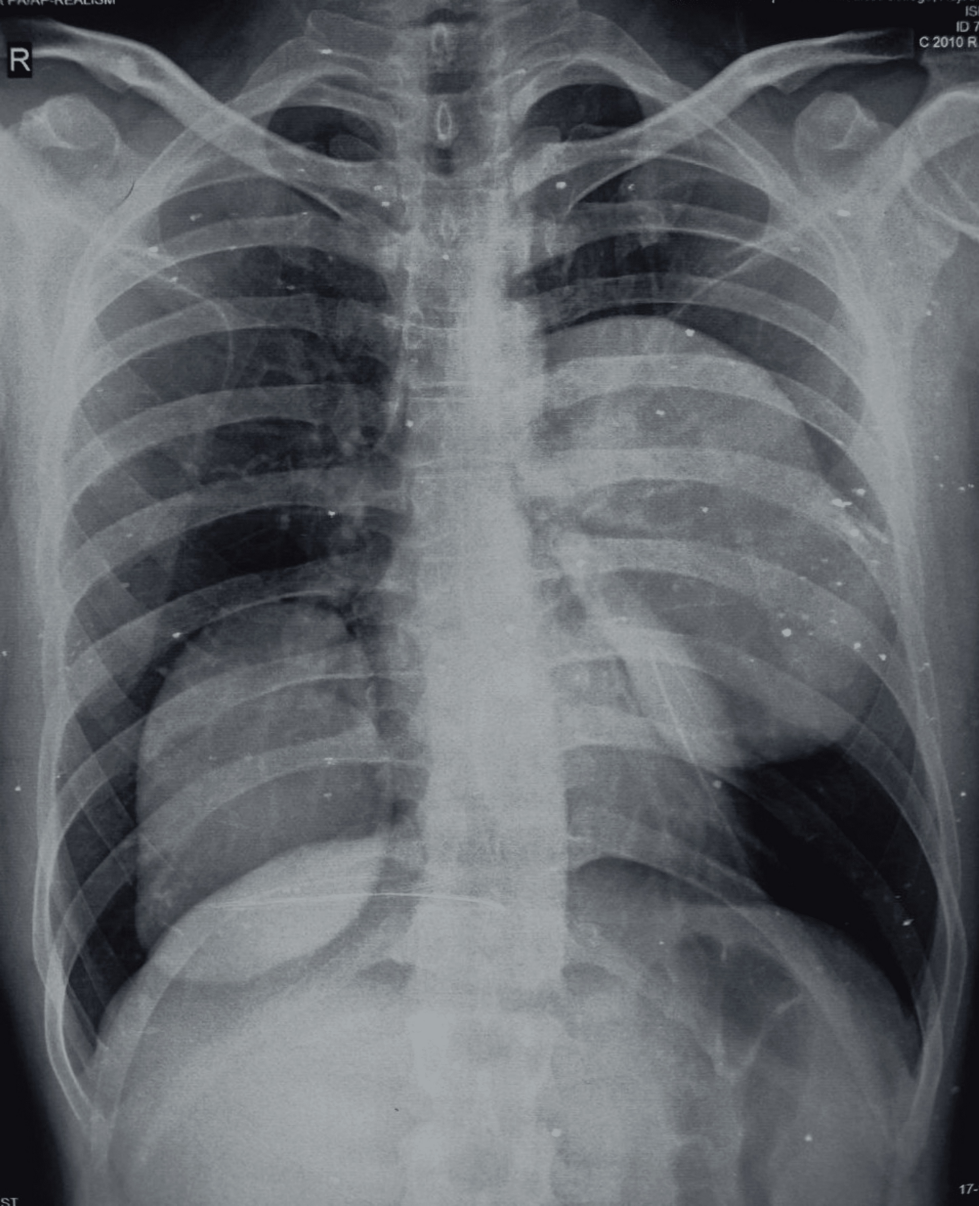
Chest X-ray showing hypodense lesions in both lobes

As part of the baseline investigations, a chest X-ray was ordered, which showed well-demarcated hypodense lesions in both lungs (Figure 1).

**Figure 2:**
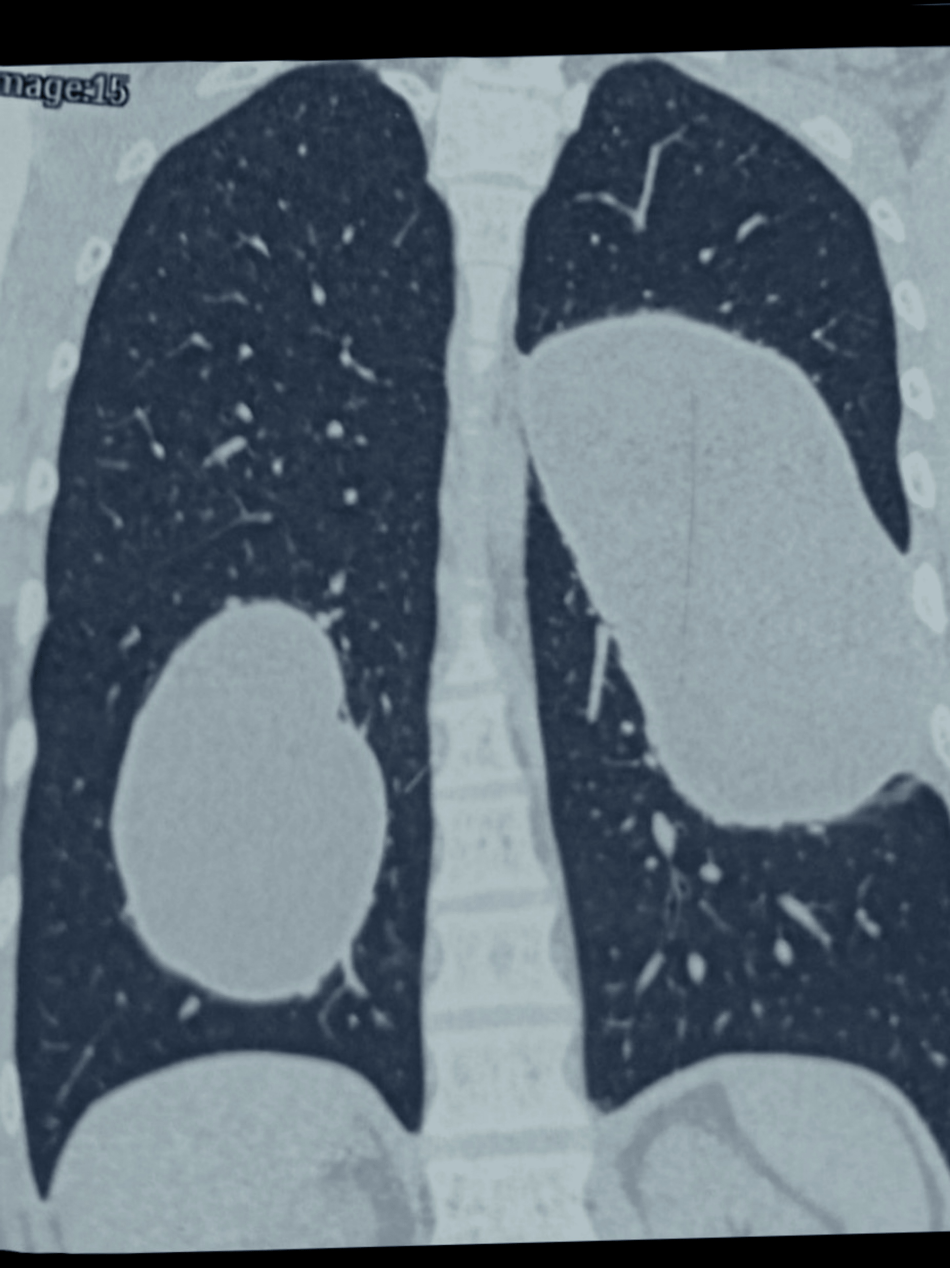
CECT chest showing hypodense, non-enhancing cystic lesions in bilateral lungs suggestive of hydatid cysts in the lungs CECT: contrast-enhanced computed tomography

Following this, a contrast-enhanced CT (CECT) of the chest was ordered, which revealed hypodense, non-enhancing cystic lesions in bilateral lungs suggestive of hydatid cysts in the lungs (Figure 2).

Given a strong suspicion of hydatid disease, Echinococcus serology was ordered, which came out positive, making the diagnosis of hydatid disease of the lungs. CECT of the abdomen was performed to evaluate for other lesions, which showed no additional lesions.

The patient was referred to cardiothoracic and vascular surgery (CTVS), where he underwent surgery to remove the primary lesions. Postoperatively, he was started on four weeks of therapy with albendazole, and the course has been uneventful since then.

## Discussion

Pulmonary hydatid disease can present independently without any visible effects on the liver, as seen in this case, and a clear history of exposure is often difficult to elicit. Different imaging modalities like chest X-rays, CT scans, and MRIs can be used to diagnose pulmonary hydatid disease [5]. Serological tests are mostly used as supporting diagnostic tools because they lack sensitivity and specificity. They can be used in follow-up to monitor disease recurrence [6].

The clinical presentation of hydatid cysts in the lungs depends on their location and size. Small and uncomplicated cysts remain clinically silent and are discovered incidentally on chest imaging. According to a study by Shehatha et al., 37% of 763 cases were found to be asymptomatic [7].

In most symptomatic patients, cough is the predominant symptom [8]. As the cyst grows in size, it can exert pressure effects on adjacent structures, leading to patients developing clinical symptoms and/or complications [4]. When these cysts grow large enough (>5 cm), they can result in bronchial compression. Complications like rupture, secondary infection, pneumothorax, and suppuration may also arise. After a cyst ruptures, patients may develop a sudden onset of chest pain, fever, cough, and hemoptysis. Some patients may also complain of a salty taste in the mouth, which is usually an indication of a ruptured cyst. The development of a hypersensitivity reaction is another dreaded complication of cyst rupture, and this reaction can range anywhere from urticaria and wheezing to a full-blown anaphylactic reaction [1,9].

As reported by Singh et al., the most common presenting symptom in up to 70% of adult patients is hemoptysis. The occurrence of hemoptysis in pediatric patients, however, is rare [10]. Pulmonary hydatid disease can result in hemoptysis via a number of mechanisms. These include pressure erosion of the bronchus, bronchial obstruction due to secondary infection, or rupture of a cyst into the bronchus. Erosion of major vessels, such as the aorta, by the cyst can lead to massive hemoptysis [11]. Secondary bacterial infection is the gravest complication of perforated cysts. Infection results in a density of over 20 Hounsfield units on a CT scan, and it can be difficult to differentiate between the infected cyst and an abscess or neoplasm [12].

Pulmonary hydatid cysts can be managed pharmacologically and/or surgically. Pharmacological treatment is targeted at small cysts, multiple cysts, patients with disseminated disease, recurrent cysts, or those with contraindications to surgery. Pharmacologic treatment is also helpful in cases where patients present with intraoperative spillage of hydatid fluid [1,13]. The drug of choice for pharmacologic treatment is albendazole [14]. Medical management may, however, not be ideal in all scenarios. Pregnant patients, patients with inactive or calcified cysts, bone marrow depression, and cysts larger than 6 cm in diameter are not suited for pharmacological treatment [15].

The gold standard of treatment for pulmonary hydatid cysts of any size is surgery. The surgical approach is mostly used for large cysts that are superficial and prone to rupture, infected cysts, cysts that lie in close proximity to vital anatomical structures, and cysts that exert a mass effect on surrounding structures [16].

## Conclusions

This case report highlights the rare occurrence of primary pulmonary hydatid disease without any effects on the liver in a 30-year-old male. It is important to keep in mind the possibility of hydatid disease in patients presenting with respiratory symptoms, especially in endemic areas. Imaging modalities like chest X-rays, CT scans, and MRIs can be used to diagnose pulmonary hydatid disease. Serological tests are mostly used as supporting diagnostic tools. Treatment involves surgical removal of the cysts, followed by medical therapy with albendazole to prevent recurrence. Early diagnosis and treatment can prevent complications and improve outcomes. Health education and improved awareness can help in the prevention and control of this disease.
